# Current Progress of Cell Therapy Clinical Trials in China in 2014–2024

**DOI:** 10.1155/bmri/1901024

**Published:** 2025-10-23

**Authors:** Wei Shi, Chunfeng Du, Zhuo Chen, Weiyi Guo, Fengshan Li, Qin Zou, Shiyin Feng, Linrui Cai, Qin Yu

**Affiliations:** ^1^ National Drug Clinical Trial Institution, West China Second Hospital, Sichuan University, Chengdu, China, scu.edu.cn; ^2^ Key Laboratory for Technical Research on Drug Products In Vitro and In Vivo Correlation, NMPA, Chengdu, China; ^3^ Children’s Medicine Key Laboratory of Sichuan Province, Chengdu, China; ^4^ Key Laboratory of Birth Defects and Related Diseases of Women and Children, Sichuan University, Ministry of Education, Chengdu, China, meb.gov.tr

**Keywords:** 2014–2024, cell therapy, China, clinical trials, drug

## Abstract

**Background:**

This paper summarizes the cell therapy clinical trials in China over the past decade to put forward suggestions for future clinical development in this field.

**Methods:**

Mainly based on the Centre for Drug Evaluation of China National Medical Products Administration (NMPA) website, a list and detailed information of cell therapy drugs in China were acquired. The annual number and basic characteristics including cell type, clinical trial phase, clinical trial status, geographical distribution, disease types, and drug target of registered drugs were summarized.

**Results:**

There have been 206 cell therapy trials between 2014 and 2024 in China. The top three cell types are T cells (51.4%, 106/206), stem cells (34.0%, 70/206), and progenitor cells (4.9%, 10/206), respectively. In terms of the phase distribution, 64.1% (132/206) were Phase I, 32.5% (67/206) were Phase II, and 3.4% (7/206) were Phase III. Currently, 86.4% (178/206) of cell therapy trials are open, 9.7% (20/206) have been completed, and the remainder are either suspended or terminated. Geographical analysis showed that 31.6% (65/206) of trials were conducted in Shanghai, 20.4% (42/206) in Beijing, and 9.2% (19/206) in Jiangsu. Twenty‐eight disease types were identified, of which lymphoma (15.5%, 32/206) was the top disease type, followed by leukemia (12.6%, 26/206) and inflammation (9.2%, 19/206). The three most common targets were CD19 (54.9%, 39/71), BCMA (9.9%, 7/71), and EGFR (5.6%, 4/71).

**Conclusions:**

Cell therapy clinical trials in China have developed rapidly in the past decade. Perhaps in the future, the direction worthy of attention could include increasing funding for Phase III clinical trials, supporting a more balanced geographical distribution of trials, and more investment in target development. With the continuous progress of technology, cell therapy is expected to become an important means to treat various diseases.

## 1. Introduction

Cell therapy clinical trials are of great significance in the field of medicine [1–3]. Cell therapy mainly includes two types: immune cell therapy and stem cell therapy. Immune cell therapy includes chimeric antigen receptor (CAR)–T cell therapy [4], tumor‐infiltrating lymphocyte (TIL) therapy [5], and natural killer (NK) cell therapy [6]. Stem cell therapy includes hematopoietic stem cell transplantation [7], mesenchymal stem cell therapy [8], and induced pluripotent stem cell (iPS) therapy [9]. Among them, CAR‐T cell therapy is a form of immunotherapy in which a patient’s own T cells are genetically engineered to express CARs, enabling them to specifically recognize and eliminate cancer cells expressing target antigens [4, 10].

In 2010, Kochenderfer et al. first reported [11] the use of CAR‐T cell therapy in a patient with advanced follicular lymphoma who experienced significant regression of the lymphoma after infusion of CD19‐targeting CAR‐T cells. Since then, CAR‐T cell therapy has shown strong potential in the treatment of hematological tumors. In 2017, the US Food and Drug Administration (FDA) approved two CAR‐T cell therapy products, Kymriah and Yescarta, for the treatment of certain types of leukemia and lymphoma. This marks the moment the cell therapy officially entered the stage of clinical application and brought new hope to cancer patients. For stem cell therapy, South Korea approved the world’s first stem cell therapy, Hearticellgram‐AMI, for the treatment of acute myocardial infarction in 2011. This approval marks a major breakthrough in the field of stem cell therapy in cardiovascular disease. Moreover, for TCR‐T cell therapy, China’s National Medical Products Administration (NMPA) approved the first TCR‐T cell therapy product, Kimmtrak, for the treatment of a specific type of uveal melanoma in 2023. Kimmtrak is a bispecific protein with the targets of glycoprotein 100 and CD3 [12]. Its TCR part targets the glycoprotein 100‐HLA‐A∗02:01 complex on cancer cells, while the anti‐CD3 part activates T cells, leading to cancer cell apoptosis. The approval of this product offers a novel treatment for this hard‐to‐treat cancer, marking an important advance in the field of cell therapy in China. These milestones (Table 1) not only demonstrate the great potential of cell therapy but also provide new directions for future medical development.

**Table 1:** Key milestones in cell therapy clinical trials.

2009	The US Food and Drug Administration (FDA) has approved the first clinical trial of stem cells to treat a genetic eye disease called retinitis pigmentosa.
2011	South Korea approved the world’s first stem cell therapy, Hearticellgram‐AMI, for the treatment of acute myocardial infarction.
2012	The world reported the first successful case of CAR‐T cell therapy, which opened a new era of CAR‐T cell therapy.
2015	The National Cancer Institute (NCI) has reported a successful case of TCR‐T cell therapy in the treatment of solid tumors for the first time.
2017	The US Food and Drug Administration (FDA) has approved two CAR‐T cell therapy products, marking the formal entry of cell therapy into the clinical phase.
2023	China’s National Medical Products Administration (NMPA) has approved the first TCR‐T cell therapy product named Kimmtrak for the treatment of a specific type of uveal melanoma.

**Figure 1:**
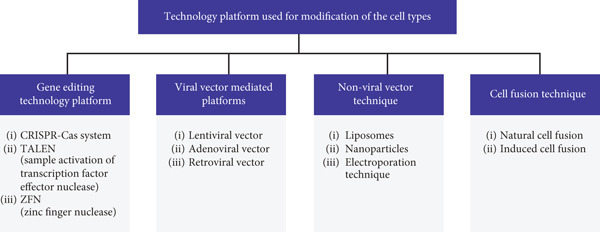
Technology platform used for modification of the cell types.

Cell therapy has emerged as a promising field in modern medicine. To understand its underlying mechanisms and potential, it is essential to first explore the cell type modification technology platforms that serve as the cornerstone. There are several technical platforms for modifying cell types (Figure 1). Gene editing technologies such as CRISPR‐Cas, TALEN, and ZFN enable precise manipulation of genes within cells. Viral vector‐mediated platforms like lentiviral, adenoviral, and retroviral vectors can introduce exogenous genes into cells, with each having its own characteristics in terms of integration, safety, and transduction efficiency. Nonviral vector techniques, including liposomes, nanoparticles, and electroporation, offer alternative ways to deliver genetic materials; liposomes are biocompatible, nanoparticles have precise targeting capabilities, and electroporation provides a simple and rapid approach. Cell fusion technologies, both natural and artificially induced, allow the combination of different cell types to create novel cellular phenotypes and functions, which have potential applications in various fields such as regenerative medicine and antibody production. These platforms enable precise manipulation and engineering of cells, thereby opening up new avenues for treating various diseases.

Phase I clinical trials focus on assessing safety and determining appropriate dosages in a small cohort; Phase II trials evaluate preliminary efficacy and further examine safety in a larger patient group; and Phase III trials are aimed at confirming long‐term efficacy while monitoring adverse effects through large‐scale, randomized controlled studies. While significant progress has been made in cell therapy clinical trials in recent years, they still face several challenges. (1) Safety issues [13]: Cell therapy may cause adverse reactions such as immune response, infection, and tumorigenesis. Rigorous quality control and monitoring are needed to ensure patient safety. (2) Effectiveness evaluation [14]: Due to the complex mechanism of cell therapy, it is difficult to evaluate the efficacy. It is necessary to establish scientific and reasonable evaluation indexes and methods. (3) High cost [15]: The high cost of preparation and implementation of cell therapy limits its wide application. Ways to reduce costs and improve access to cell therapy need to be explored.

So, the purposes for summarizing the current progress of cell therapy clinical trials are (1) to provide an overview of the research status in China over the past decade and (2) to put forward recommendations to further promote the development of cell therapy drugs and provide data support for industries and research institutions.

## 2. Methods

### 2.1. Data Collection

Data involved in this study were acquired from the website of the Centre for Drug Evaluation, China NMPA—a website officially designated and authorized for registration use. We systematically searched this website for registered cell therapy trials in China. Cell therapy clinical trial data for 2024 are available up to July 9, 2024.

### 2.2. Inclusion and Exclusion Criteria

Inclusion criteria: we included clinical trials for treating various diseases through cell therapy. Through the search terms “Cell” and “Biological product,” there would be a certain selection bias. Trials that contain the word “cell” but are not cell therapy may be mistakenly included.

So the exclusion criteria were as follows: (1) We excluded clinical trials of noncellular therapeutics. (2) We excluded trials whose trial status was BE trial and clinical trials whose phase was not specified. (3) We excluded vaccine clinical trials.

### 2.3. Statistical Analysis

SPSS 26.0 software was used for all statistical analyses. Frequency and percentage were used for the characteristic description of cell therapy clinical trials.

### 2.4. Selection and General Characteristics of Studies

**Figure 2:**
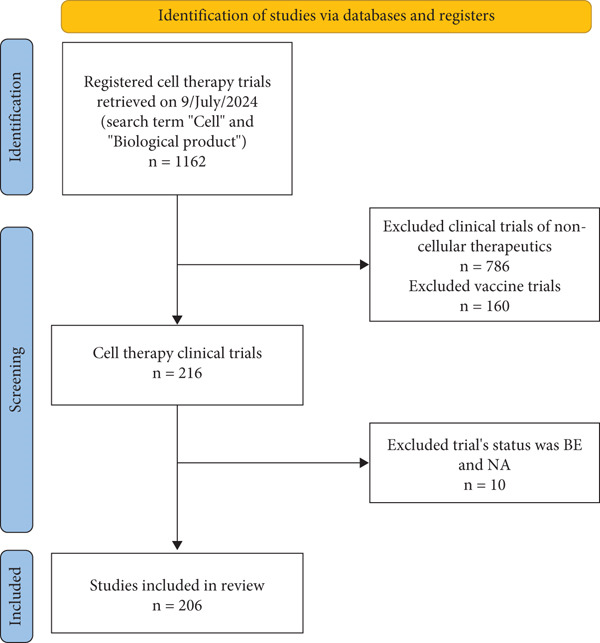
Flowchart for the selection and data sorting of cell therapy clinical trials registered on drug clinical trial registration and information publicity platform considered in this study.

In the final analysis, only 206 cell therapy clinical trials were included. A total of 956 trials were excluded, including noncellular therapeutic trials (*n* = 786), vaccine trials (*n* = 160), and trials with status BE/NA (*n* = 10), as shown in Figure 2. We used the PRISMA reporting guidelines.

## 3. Results

**Figure 3:**
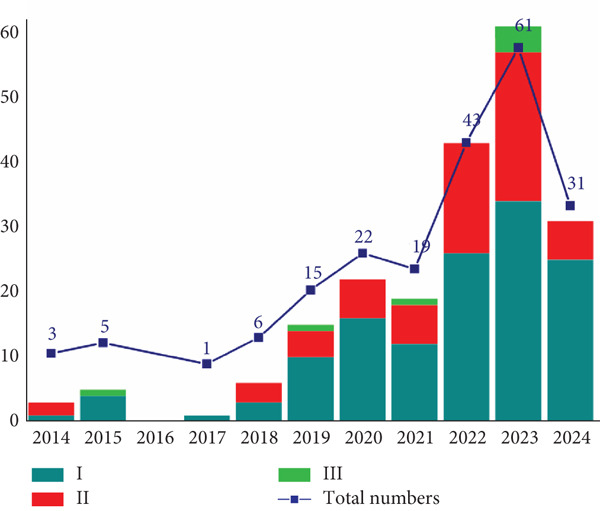
Analysis of numbers of clinical trials on cell therapy by year and clinical stage. Data were obtained from the drug clinical trial registration and information publicity platform.

In recent years, there have been 206 trials of cell therapy between 2014 and 2024 (Table 2 and Figure 3). Currently, the types of cell therapies entering clinical trials are T cell, stem cell, progenitor cell, and so on. In 2014–2024, the top three cell types are T cells (51.4%, 106/206), stem cells (34.0%, 70/206), and progenitor cells (4.9%, 10/206), respectively. With the development of cell therapy clinical trials, the number of trials peaked in 2022 and 2023 (the 2024 clinical trial data is incomplete as of July 2024). In terms of the phase distribution, 64.1% (132/206) were Phase I, 32.5% (67/206) were Phase II, and 3.4% (7/206) were Phase III trials in China. In the Phase I clinical trials, the T cell type (53.7%, 71/132) was the most, followed by the stem cell type (31.8%, 42/132). The top two Phase II clinical trial types were T cell trials (52.2%, 35/67) and stem cell trials (37.3%, 25/67). Stem cells (42.9%, 3/7) and progenitor cells (42.9%, 3/7) were the top two types in the Phase III clinical trials. We have also calculated the average duration of completed trials across different phases (Phases I, II, and III) and different types of cell therapy, as well as the current duration of ongoing trials. For completed clinical trials, in Phase I, three types of cell therapy have completed clinical trials, with durations as follows: stem cells (7 years, 2 trials), T cells (4.5 years, 8 trials), and undifferentiated immune cells (4 years, 1 trial). In Phase II, only one type of cell therapy has completed clinical trials: stem cells, with a duration of 8 years (3 trials). No cell therapy types have completed clinical trials in Phase III. For ongoing cell therapy clinical trials, their duration ranges from 1 to 9 years.

**Table 2:** Different clinical trial stage distributions for cell therapy.

**Cell type**	**Phase I**	**Average duration of Phase I (year)**		**Phase II**	**Average duration of Phase II (year)**		**Phase III**	**Average duration of Phase III (year)**	**Total**
T cell	71 (53.7)	2.38^a^	4.5^b^	35 (52.2)	2.71^a^		0 (0.0)	0	106 (51.5)
Stem cell	42 (31.8)	1.52^a^	7^b^	25 (37.3)	1.35^a^	8^b^	3 (42.9)	1^a^	70 (34.0)
Progenitor cell	6 (4.5)	1.33^a^		1 (1.5)	1^a^		3 (42.9)	1^a^	10 (4.8)
Unsubdivided immune cells	2 (1.5)	1^a^	4^b^	3 (4.5)	1.33^a^		0 (0.0)	0	5 (2.4)
NK cell	5 (3.8)	1.25^a^		0 (0.0)	0		0 (0.0)	0	5 (2.4)
DC cell	3 (2.3)	1^a^		0 (0.0)	0		1 (14.2)	9^a^	4 (1.9)
Cardiomyocyte	1 (0.8)	1^a^		1 (1.5)	1^a^		0 (0.0)	0	2 (1.0)
HD003 cell	1 (0.8)	1^a^		1 (1.5)	1^a^		0 (0.0)	0	2 (1.0)
RAK cell	1 (0.8)	7^a^		1 (1.5)	1^a^		0 (0.0)	0	2 (1.0)
Total	132	—		67	—		7	—	206

^a^The figure represents the average duration of the trials with the status of still ongoing.

^b^The figure represents the average duration of the trials with the status of completed.

**Figure 4:**
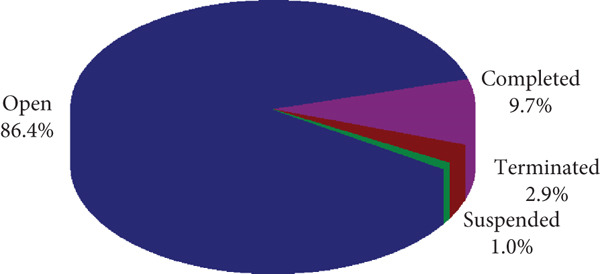
The current status of cell therapy clinical trials in China. Data were obtained from the drug clinical trial registration and information publicity platform.

**Figure 5:**
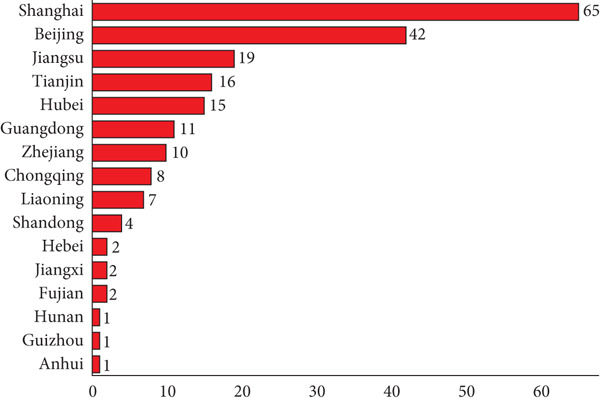
Geographical distribution of cell therapy drugs in China. Data were obtained from the drug clinical trial registration and information publicity platform.

Currently, 86.4% (178/206) of cell therapy trials are open, 9.7% (20/206) have been completed, and the remainder are either suspended or terminated (Figure 4). Geographical analysis showed that 31.6% (65/206) of these trials were conducted in Shanghai, 20.4% (42/206) in Beijing, and 9.2% (19/206) in Jiangsu (Figure 5). These three provinces are the main ones where clinical trials of cell therapy are conducted.

**Figure 6:**
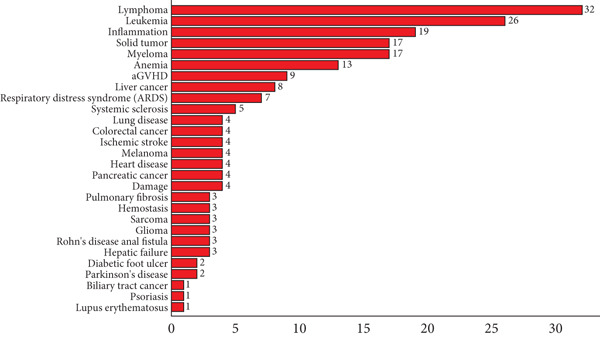
Number of cell therapy clinical trials on different disease types. Data were obtained from the drug clinical trial registration and information publicity platform.

Cell therapy clinical trials cover a variety of disease types, mainly including the following categories: blood system diseases (leukemia, lymphoma, and multiple myeloma), immune system diseases (rheumatoid arthritis, systemic lupus), neurological diseases (Parkinson’s disease, Alzheimer’s disease, and spinal cord injury), cardiovascular system diseases (myocardial infarction, heart failure), solid tumors (lung, liver, breast, and pancreatic), and other diseases (diabetes, osteoarthritis, burns, and trauma). For the cell therapy trials initiated in China, 28 disease types were identified (Figure 6). Lymphoma (16%, 32/206) continues to be the top disease type with the most cell clinical trials, followed by leukemia (13%, 26/206) and inflammation (9%, 19/206).

**Figure 7:**
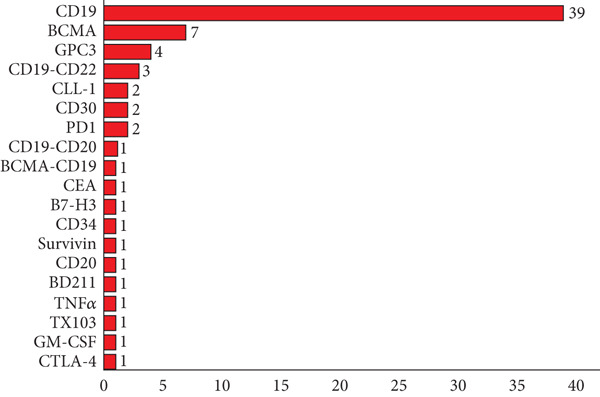
Target distribution of cell therapy drugs in China. Data were obtained from drug clinical trial registration and information publicity platform. (Special note: there are some clinical trials that have not published targets.)

As shown in Figure 7, 19 targets have been explored in China in the past decade (special note: there are some clinical trials that have not published targets). The three most common targets developed in China were CD19 (54.9%, 39/71), BCMA (9.9%, 7/71), and EGFR (5.6%, 4/71). Multitarget cell therapies were CD19‐CD22 (4.2%, 3/71), CD19‐CD20 (1.4%, 1/71), and BCMA‐CD19 (1.4%, 1/71).

## 4. Discussion

Cell therapy clinical trials in China have been developing rapidly in recent years. The number of cell therapy clinical trials in China is growing rapidly, peaking in 2022 and 2023 at 43 and 61, respectively. In addition, cell types, the indications, and targets for cell therapy clinical trials in China are diverse. Moreover, the geographical distribution of cell therapy clinical trials in China is extensive, covering 16 provinces. The top three provinces are Shanghai, Beijing, and Jiangsu.

The number of Phase III cell therapy clinical trials increased in recent years, but the proportion was still low (3.4%, 7/206). Many drugs are eliminated due to reasons such as insignificant efficacy or safety issues after the screening of Phase I and Phase II, and fewer drugs can enter into Phase III. Moreover, the progression of Phase III cell therapy trials is influenced by multiple factors, and resource allocation (including funding) is frequently cited in industry reports as a potential contributing factor [16, 17]. Perhaps in the future, the direction worthy of attention could include increasing funding for Phase III clinical trials and improving the trial design and management to accelerate the process of Phase III clinical trials.

While immune cell and stem cell therapies appear similar in Phase III progression, their clinical development diverges fundamentally [18]. In Phase I, immune cell therapy (e.g., CAR‐T) focuses on acute adverse reactions (e.g., cytokine release syndrome) due to rapid immune activation [19], whereas stem cell therapy prioritizes long‐term safety (e.g., differentiation abnormalities) [20]. Phase II trials see immune cell therapy emphasizing objective response rates in specific malignancies, while stem cell therapy focuses on functional recovery metrics (e.g., tissue regeneration) [20]. Immune cell therapy follows a “depth‐first” path—succeeding in narrow fields like hematologic cancers but struggling with solid tumors [21]—while stem cell therapy adopts a “breadth‐first” strategy, with trials across neurology, cardiology, etc., yet facing higher late‐phase attrition. Thus, their similar Phase III presence masks a core difference: immune cell therapy is a precision weapon seeking new targets, stem cell therapy a repair tool seeking definitive applications [22].

This study presents the quantitative analysis of clinical trial durations in cell therapies in China. Results show that the average duration of completed trials varies by phase and therapy type: for the completed trials, the duration of Phases I–II was 4.5–8 years; no Phase III trials have been completed. Ongoing trials have already lasted 1–9 years, indicating that most programs remain in extended development cycles. Consistent with global trends, cell therapy development is universally long term—FDA‐approved CAR‐Ts take 5–10 years from concept to approval [23], and EU ATMPs face similarly protracted paths—aligning with this study’s findings and underscoring high time and capital demands [24]. A key difference lies in Phase III efficiency: China’s lack of completed Phase III cell therapies and few ongoing ones may reflect challenges in scaling funding, managing multinational trials, and navigating the “valley of death.” For investors, this means cell therapy requires long‐term capital, with financing plans based on 5–10‐year benchmarks. Encouraging long‐term investment via policy and sophisticated capital instruments is critical to boosting R&D efficiency and bringing products to market.

The geographical distribution of cell therapy clinical trials is uneven, mainly concentrated in Shanghai, Beijing, and Jiangsu. It may lead to insufficient representativeness of trial data, affect the efficiency of clinical trials (due to fierce competition in subject recruitment), and result in unequal benefits for patients. The reason is speculated to be the concentration of medical resources, strong scientific research capabilities, and developed economies in these places. In the future, policy guidance and support, regional collaborative cooperation, and balanced allocation of medical resources may be considered.

Limited targets have been reported in clinical trials of cell therapy in China. In China, the three most commonly developed targets are CD19, BCMA, and EGFR, with CD19 being the most prevalent, followed by BCMA and then EGFR. It is speculated that the reasons may be the target selection limited by safety issues, technical limitations, and the disconnect between basic research and clinical translation. In the future, we may consider deepening genomics and proteomics research, as well as promoting the development of new target screening technologies. We believe that with the in‐depth study of cell biology and disease mechanisms, new cell therapeutic targets are constantly being discovered to provide more options for disease treatment.

Cell therapy clinical trials in China provide a new therapeutic approach and hope for some diseases that are not effective with traditional therapies, such as some cancers and autoimmune diseases [25]. China has made continuous innovations and breakthroughs in cell therapy technology [13], such as the application of gene editing technology and new cell culture technology, which have improved the effectiveness and safety of cell therapy and also reduced the cost and production difficulty. In recent years, cell therapy has demonstrated remarkable clinical outcomes, with several CAR‐T cell therapies successfully transitioning from clinical trials to approved treatments. Two notable examples include (1) ciltacabtagene autoleucel (cilta‐cel, developed by Legend Biotech)—a B cell maturation antigen (BCMA)‐targeting CAR‐T therapy. Approved by NMPA in 2024 (Approval Number S20240038), cilta‐cel was supported by data from the Phase II CARTIFAN‐1 trial (CTR20181007), which demonstrated its efficacy in treating relapsed or refractory multiple myeloma. (2) Zevor‐cel (developed by CARsgen Therapeutics) is another BCMA‐directed CAR‐T therapy. It received NMPA approval in 2024 (Approval Number S20240006) based on results from the open‐label, single‐arm, multicenter Phase II LUMMICAR STUDY 1 trial (NCT03975907), establishing its clinical benefit in multiple myeloma patients. Besides, in CRISPR‐Cas9 applications, Chinese researchers have used it to edit genes in T cells for enhanced antitumor responses in cancer immunotherapy and to correct genetic defects in hematopoietic stem cells for treating blood disorders [26]. These approvals highlight the rapid progress and therapeutic potential of CAR‐T cell therapies in hematologic malignancies. In addition, China has a large population, a rich variety of diseases, and a large number of patients who have a demand for innovative therapeutic methods, providing a broad market and application scenarios for cell therapy clinical trials. Perhaps in the future, we can realize personalized treatment of cell therapy [27, 28].

Compared with the United States and the European Union, China has certain similarities in the progress of target indications and molecular development in cell therapy. (1) Focus on hot targets: In the field of immunotherapy, especially CAR‐T cell therapy, all three regions have focused on some popular targets. For example, the CD19 target has made significant progress in the treatment of blood tumors such as B cell lymphoma and is one of the most common targets in currently approved CAR‐T drugs [29]. (2) Emphasis on hematological malignancies: Diseases such as multiple myeloma, lymphoma, and leukemia all have multiple cell therapy drugs in the R&D or approved stage. For instance, many drugs like zevorcabtagene autoleucel in China and Breyanzi in the United States have been approved for the treatment of hematological tumors [30]. (3) Exploration in solid tumors: All regions are actively conducting cell therapy research for solid tumors and working hard to overcome problems such as the difficulty in finding specific antigens for solid tumors and the immunosuppressive tumor microenvironment [31]. However, there are also certain differences in target indication expansion among China, the United States, and the European Union. For China, in addition to common targets for hematological tumors, there has been an increase in trials for some less common targets in solid tumors, such as CLDN18.2, HER2, and MSLN in recent years [32]. For the United States, the United States is relatively more extensive and advanced in target indication expansion. For example, gene therapies for diseases such as metachromatic leukodystrophy and DMD gene mutations have been approved for marketing. For the European Union, the EU is relatively conservative in the selection of targets for immunotherapy and pays more attention to targets and indications that have been verified in other regions.

Only cell therapy trials for registration purposes were included, while investigator‐initiated trials were not involved due to data availability restrictions; there may be some limitations in our study. We just made an overall description of cell therapy trials in China from a macro statistical point of view, so there might be a lack of in‐depth focus.

## 5. Conclusion

In conclusion, great progress has been made in China for cell therapy clinical trials in recent years. It is an area of research full of challenges and opportunities. Increasing funding for Phase III clinical trials, supporting a more balanced geographical distribution of clinical trials, and more investment in target development could be the direction worthy of attention in the future. Through continuous efforts and innovation, it is expected to bring new therapeutic hope to many patients.

## Ethics Statement

The authors have nothing to report.

## Consent

The authors have nothing to report.

## Disclosure

All authors read and approved the final manuscript.

## Conflicts of Interest

The authors declare no conflicts of interest.

## Author Contributions

Qin Yu designed the study. Wei Shi, Chunfeng Du, Zhuo Chen, Weiyi Guo, and Fengshan Li screened the literature. Qin Zou, Shiyin Feng, and Linrui Cai extracted the data. Wei Shi analyzed the data and wrote the manuscript. Qin Yu contributed to the critical revision.

## Funding

No funding was received for this manuscript.

## Data Availability

The datasets used and/or analyzed during the current study are available from the corresponding author on reasonable request.
